# Development of Post-Traumatic Stress Disorder (PTSD) in Students of The Medical University Under The Martial Law in Ukraine

**DOI:** 10.1192/j.eurpsy.2026.11872

**Published:** 2026-07-31

**Authors:** O. M. Pityk, N. Karbovskyi, A. Romaniv

**Affiliations:** 1 Department of Psychiatry, Narcology and Medical Psychology; 2 Ivano-Frankivsk National Medical University, Ivano-Frankivsk , Ukraine

## Abstract

**Introduction:**

The situation that has been going on in Ukraine since February 2022 has led to catastrophic consequences. This forces neuroscientists and, including psychiatrists, to turn to the study of the impact of all these events on the mental health of the population of Ukraine.

The subjective psychological impact of a traumatic event is closely associated with the extent to which the event disrupts an individual’s foundational beliefs about the world and the self, commonly referred to in psychology as “core assumptions.” These assumptions function as protective mechanisms, enabling individuals to mitigate anxiety concerning potential threats.

**Objectives:**

Students of medical universities, studying in the conditions of a military conflict, encountered an increased psychological load, which affects their psychological resilience and ability to adapt to stressful situations. As the war continues for more than two years, this has resulted in the central nervous system of young adults being in a state of chronic stress. The mental state of future doctors can determine their ability to provide high-quality medical care in the future (influence the ability to make balanced decisions, an adequate diagnostic process, the quality of forming a high-quality complex treatment protocol), so research on the development of PTSD is important for improving the quality of medical practice.

**Methods:**

A survey was conducted among 102 respondents of the Ivano-Frankivsk National Medical University of 1-6 courses, all specialties according to the PCL-5 methodology. PCL-5 methodology. involves two approaches to the interpretation of the results.

**Results:**

We examined 102 students of the Ivano-Frankivsk National Medical University aged 18 to 23. Among them, 17 (16.67%) were men and 85 were women (83.33%). The number of respondents who scored more than 33 points is 39 people (6 men and 33 women), which is 38.2% (5.9% and 32.3%, respectively) of the respondents. A positive result was found in 5 students of the 1st year (4.9%), 6 students of the 2nd year (5.88%), 14 students of the 3rd year (13.73%), 7 students of the 4th year (6.86%), 5 students 5th year (4.9%), 2 students of 6th year (1.96%).

**Conclusions:**

Research on the mental health of medical students under martial law is necessary, which creates an important scientific and practical problem in understanding and effectively managing this issue.

Thus, the mental state of medical university students living in a country under martial law requires further thorough research into the risks of developing PTSD in order to develop effective protocols for diagnosis, treatment, rehabilitation and possible methods of psychoprophylaxis.

**Disclosure of Interest:**

None Declared

